# Rearfoot striking is associated with lower modeled ACL loading during overground running: a musculoskeletal modeling study

**DOI:** 10.3389/fspor.2026.1913363

**Published:** 2026-08-06

**Authors:** Sungmin Kim, Jeheon Moon, Wing-Kai Lam

**Affiliations:** 1 Department of Sport Science, Kongju National University, Gongju, Chungnam, Republic of Korea; 2 Department of Physical Education, Korea National University of Education, Cheongju, Chungbuk, Republic of Korea; 3 Department of Sports and Health Sciences, Academy of Wellness and Human Development, Hong Kong SAR, China; 4 Centre for Exercise Science and Medicine (CESAME), Hong Kong Baptist University, Hong Kong SAR, China

**Keywords:** computational simulation, footstrike angle, GRF, joint loading, knee ligament

## Abstract

**Background:**

The purpose of this study was to examine knee ligament forces and conventional kinematic and kinetic variables across forefoot (FFS), midfoot (MFS), and rearfoot (RFS) strikes whilst controlling for running speed.

**Methods:**

Twenty healthy male participants performed overground running at 4.3 ± 0.2 m/s in each of the strikes. Joint kinematics, moments, ground reaction forces (GRFs), and cruciate ligament forces were determined using musculoskeletal modeling.

**Results:**

Results indicated that FFS exhibited significantly shorter contact times and smaller knee flexion angles compared to MFS and RFS (*p* < .05). Furthermore, FFS produced a higher peak vertical GRF than peaks observed in MFS and RFS (*p* < .05). Notably, ACL loading (both peak force and impulse) was significantly lower in RFS compared to FFS and MFS (*p* < .05), whereas PCL loading showed no significant differences across patterns. Additionally, vertical GRF demonstrated moderate-to-strong positive correlations with peak forces and impulses of the ACL (*r* = .558–.845, *p* < .01), whereas no significant correlations were found for the PCL.

**Conclusion:**

These findings suggest that the reduced ACL loading in RFS may be linked to its unique GRF profile. Conversely, higher ACL loads associated with FFS were observed under the tested straight-line running condition. Understanding these ligament mechanics could provide valuable insights for future research on injury-prevention strategies and rehabilitation protocols.

## Introduction

Footstrike patterns for forefoot (FFS), midfoot (MFS), and rearfoot strike (RFS) critically alter lower-limb loading profiles during running (1–3). While most studies have predominantly focused on macro-level metrics such as external knee joint loading, patellofemoral loading, and tibial impact loading (4, 5), less attention has been paid to internal tissue-level responses, particularly ACL and PCL ligament loading within the knee (6, 7). Since these internal loads would be associated with pain and tissue damage, investigating tissue-level mechanics across different footstrike patterns is essential to clarify specific knee injury pathways (8).

Running imposes substantially greater impact forces compared with walking, increasing demands on knee structures including the meniscus, anterior cruciate ligament **(**ACL), posterior cruciate ligament (PCL), medial collateral ligament (MCL), and lateral collateral ligament (LCL) (9). Notably, up to one-third of meniscus injuries occur concurrently with cruciate ligament injuries (10, 11). Although cruciate ligament ruptures often arise from high-risk maneuvers such as lateral cutting or pivoting (12, 13), investigating straight-line running is highly essential as it represents the most fundamental and repetitive movement task in running-related training. Epidemiological evidence indicates that the knee is the most common site for lower extremity running injuries, and that among runners with greater weekly training distance and a history of previous injury are strongly linked to increased injury risk (14). During straight-line running, the knee joint is subjected to millions of repetitive loading cycles annually; even submaximal anterior-posterior shear forces can lead to cumulative micro-trauma and mechanical fatigue of the ACL and PCL as they continuously resist tibiofemoral translation following foot contact (15–17). Over prolonged running, alterations in frontal-plane and transverse-plane moments, combined with fatigue-induced changes in mechanics, may further increase ligament loads (18, 19).

Since footstrike angle (FSA) affects lower limb posture at contact, it has potential to modulate ligament forces within the knee (20). However, despite extensive research on footstrike patterns and external biomechanics, the relationships among footstrike pattern, internal knee-ligament loading, and injury risk remain insufficiently resolved (21–23). Quantifying tissue-level mechanics including ligament force responses can offer essential insights into how different footstrike patterns influence knee loading and long-term tissue health.

To examine the ligament loading under different footstrike, we incorporated human ligament properties (e.g., force- length behavior, strain rate, and elasticity) into an OpenSim Gait2392-based musculoskeletal model for determining variables related to muscle-tendon mechanics and ligament force behavior, which were consistent with previous simulation-based running analysis (24–26). The purpose of this study was to examine knee ligament forces as well as conventional kinematic and kinetic variables across FFS, MFS, and RFS whilst controlling for running speed. Based on recent evidence, we hypothesized that RFS would exhibit higher external knee joint loads and that FFS may not proportionally reduce internal ligament loading (21, 27, 28). Findings from this work may inform clinical rehabilitation strategies and running-technique retraining for individuals with knee pathology.

## Methods

### Participants

Twenty male participants (age: 23.58 ± 3.13 years, height: 1.75 ± 0.05 m, mass: 68.2 ± 5.46 kg) were recruited for this study, which followed a sample size comparable to previous work (29). *A priori* sample size estimation was performed using a t-test model in G*Power 3.1 based on pilot data from four participants. Inclusion criteria were: (i) right-foot dominance; (ii) engagement in regular physical activity for 3–4 h per day; (iii) no history of morbidity or musculoskeletal injury within the past six months (4, 29). All participants met these criteria, and all data was anonymized in accordance with ethical guidelines. The study protocol was approved by the Institutional Review Board of Korea National University of Education and conducted in accordance with the Declaration of Helsinki (KNUE-202401-BM-0432-01).

### Apparatus

A seventeen-camera motion capture system (Oqus 7+, Qualisys, Sweden, sampled at 150 Hz) synchronized with a force plate (Type 9260AA6; Kistler, Switzerland, sampled at 1,500 Hz) was used to collect the kinematic and kinetic data. Forty reflective markers (12 mm diameter) were placed on anatomical landmarks of the upper and lower extremities to define our musculoskeletal model (30, 31). Landmarks included ASIS, PSIS, sacrum, joint centers of the shoulder, elbow, hip, knee, and ankle joints, with additional triad of tracking markers on the head, trunk, upper arms, lower arms, thighs, shanks, and feet (30–32).

### Procedure

All participants wore tight-fit apparels and identical running shoes (Revolution 7 EasyOn, Nike, USA) to ensure consistent placement of reflective markers. Each participant began running from a starting point 5 m posterior to the force plate and was instructed to maintain a speed of 4.3 ± 0.2 m/s (33, 34). This speed was selected as it is suitable for sustaining initial running pace and assessing injury risk (34). Participants were provided sufficient familiarization time and were instructed to maintain a constant velocity throughout each trial (35). They were instructed to land on the force plate with the right foot without accelerating or decelerating. In this study, only right-leg data were analyzed as it represented the dominant limb for all participants. Running speed was monitored using a pair of timing gates (Witty, Microgate, Italy) positioned along the runway at 5 m intervals. The first gate was placed 2.5 m before the center of the force plate and the second gate 2.5 m after. Speed measurement began as participants passed the first gate and ended at the second.

**Figure 1:**
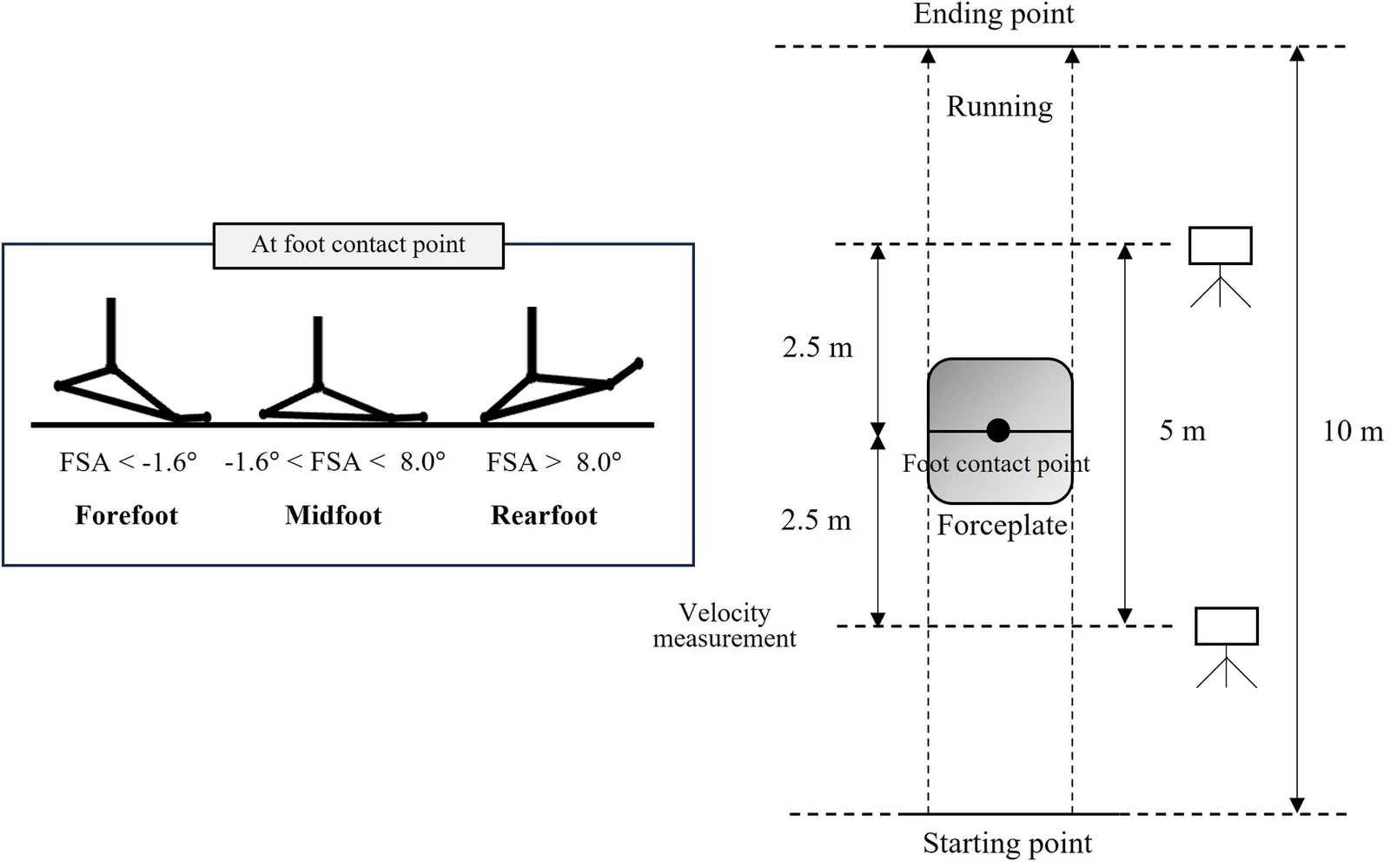
The left panel displays the classification of the three experimentally imposed footstrike patterns determined by the footstrike angle (FSA) at initial contact: forefoot strike (FFS, $\mathrm{FSA}<-1.6^{\circ }$), midfoot strike (MFS, $-1.6^{\circ }\leq \mathrm{FSA}\leq 8.0^{\circ }$), and rearfoot strike (RFS, $\mathrm{FSA}>8.0^{\circ }$). The right panel illustrates the schematic layout of the 10 m overground running runway, including the starting point, ending point, and the centrally embedded forceplate used to capture GRF (ground reaction forces) at the foot contact point.

Participants performed running trials using three footstrike patterns as defined in previous studies (36). Since participants were not selected based on their habitual footstrike preferences, they were explicitly instructed to alter their running mechanics to achieve each target pattern during the trials. FFS was identified by a footstrike angle (FSA) < −1.6°, where the forefoot contacted the ground before the heel. MFS was identified by an FSA between −1.6° and 8.0°, characterized by near-simultaneous forefoot and rearfoot contact. RFS was defined by an FSA > 8.0°, where the heel contacted the ground first (Figure 1). Additionally, all participants were provided with a sufficient familiarization and practice period prior to the formal data collection to ensure they could consistently adapt to and execute each target footstrike pattern.

The same researcher reviewed the classification of successful and unsuccessful data immediately after each running trial, using marker-based foot kinematics to verify FSA. Ambiguous cases were discussed by two experimenters to reach a consensus on final classification. Five successful trials were recorded for each footstrike pattern. A trial was considered successfully if (i) the entire right foot contacted the force plate and (ii) the footstrike pattern was confirmed via retrospective inspection of FSA. Trials were discarded if (i) participants did not maintain the target speed range, (ii) the footstrike pattern did not meet the predefined criteria, or (iii) the foot did not fully contact the force plate. Unsuccessful trials were immediately discarded from the analysis, and additional trials were performed until five successful repetitions were achieved for each condition. Consequently, the total number of attempts differed across conditions due to varying task difficulties. The order of footstrike conditions was randomized using random number generator in the MATLAB (R2016b, MathWorks, USA). Each participant completed five trials per footstrike condition with a 3 min rest between footstrike conditions to mitigate any confounding fatigue effects arising from repeated attempts. In total, 15 successful trials were collected per participant (5 trials × 3 conditions) consistent with the previous running protocol (37). The success rate of the FFS, MFS, and RFS conditions were 100%, 78.2%, and 95%, respectively.

### Data processing

A fourth-order Butterworth low-pass filter with a cut-off frequency of 15 Hz was applied to the raw data of kinematics and GRF (38). Based on the classified footstrike patterns, key biomechanics parameters including cadence, step length, contact time, peak GRF, knee joint angles and moments, and ACL and PCL forces were calculated for subsequent analysis. Joint kinetic data was down-sampled prior to computation to match the sampling frequency of the kinematic signals.

#### Conventional kinematic and kinetic variables

Cadence was defined as the number steps per minute, calculated from the 60 s window divided by step time (i.e., consecutive left-foot contacts). Contact time was defined as the duration from initial contact to toe-off of the right foot remained on the ground. Step length was measured as the anterior-posterior distance between the heel (or rear of the forefoot) of the right and left feet at successive foot contacts.

Right-knee kinematic data were converted from quaternions to Euler angles (rotation sequence: XYZ) to permit comparison with the 3D motion-analysis output (39). Joint moments were computed via inverse dynamics in Visual3D (C-Motion, Rockwille, MD, USA) and normalized to each participant's height and body mass (29, 40). All variables were reported as peak positive (+) or negative (−) values according to the right-hand rule from initial contact to peak knee flexion (+X: flexion, −X: extension, +Y: adduction, −Y: abduction, +Z: internal rotation, −Z: external rotation) (29).

GRF during stance was normalized to body weight (BW) (41). Forefoot contact was defined as the period from the vertical GRF exceeding 10 N following the right-foot initial ground contact.

#### Knee ligament modeling

**Figure 2:**
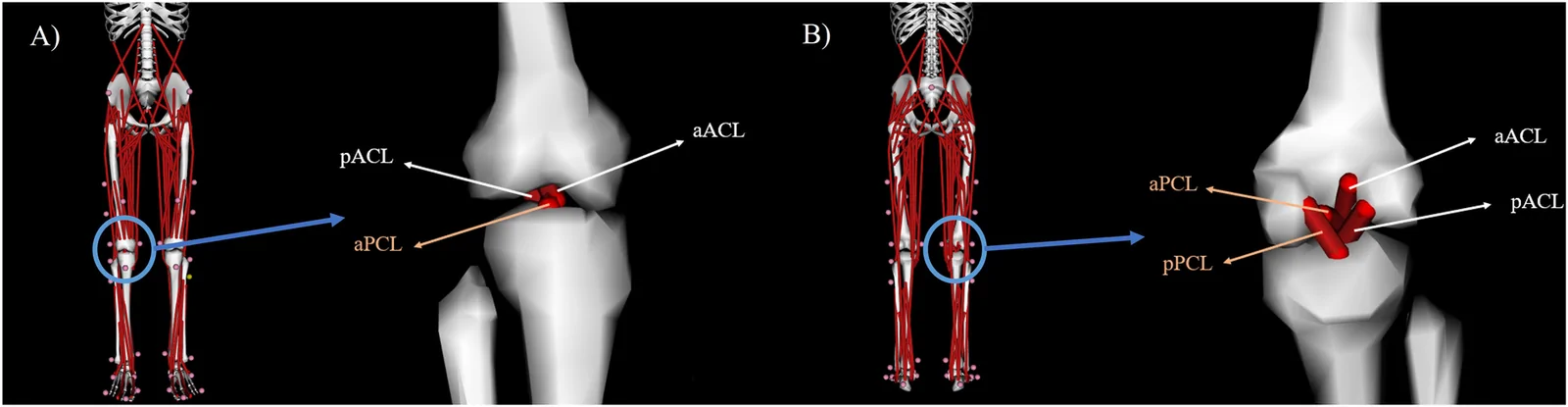
Knee cruciate ligament representation in the musculoskeletal model. **(A)** Anterior view and **(B)** posterior view of the full-body model with right knee insets highlighting the four functional ligament bundles (aACL, pACL, aPCL, and pPCL) *aACL/pACL, anterior/posterior strands of the anterior cruciate ligament; aPCL/pPCL, anterior/posterior strands of the posterior cruciate ligament.

OpenSim software (version 3.3, Stanford University, CA, USA) was used to model knee ligaments and musculotendon dynamics. The software estimates internal biomechanical variables based on input kinematic, kinetic, and musculoskeletal data (24, 25). We employed the Gait2392 musculoskeletal model developed in previous work (42), which incorporates 14 body segments, 23 degrees of freedom, 92 musculotendon actuators, 9 bones, and 15 joints (43). To quantify knee ligament loading across footstrike patterns, both the ACL and PCL were represented by two functional strands (anterior and posterior) (42). In total, four ligament strands were implemented to capture the geometric and mechanical properties of the cruciate ligaments, with ACL and PCL each modeled as distinct anterior and posterior bundles (44, 45) (Figure 2 and Table 1).

**Table 1:** ACLs and PCLs properties.

Ligaments	Coordinate (position)						Resting length (mm)	Max isometric force (F_0_, N)
	Origin (x, y, z)(mm)			Insertion point (x, y, z)(mm)				
aACL	−0.72	−40.04	0.41	1.66	−3.00	−0.07	32.3	1,500
pACL	−1.50	−40.98	1.00	0.25	−3.25	0.00	24.7	1,600
aPCL	−0.87	−41.34	−0.93	−2.05	−3.31	0.33	25.8	2,600
pPCL	−1.59	−40.57	−1.63	−1.47	−3.18	−0.41	25.2	1,900

Coordinates are defined within the local anatomical reference frames of the femur (for origins) and tibia (for insertions) in millimeters (mm). Resting length values represent the baseline parameters scaled from the reference model (48).

##### OpenSIM data processing

**Figure 3:**
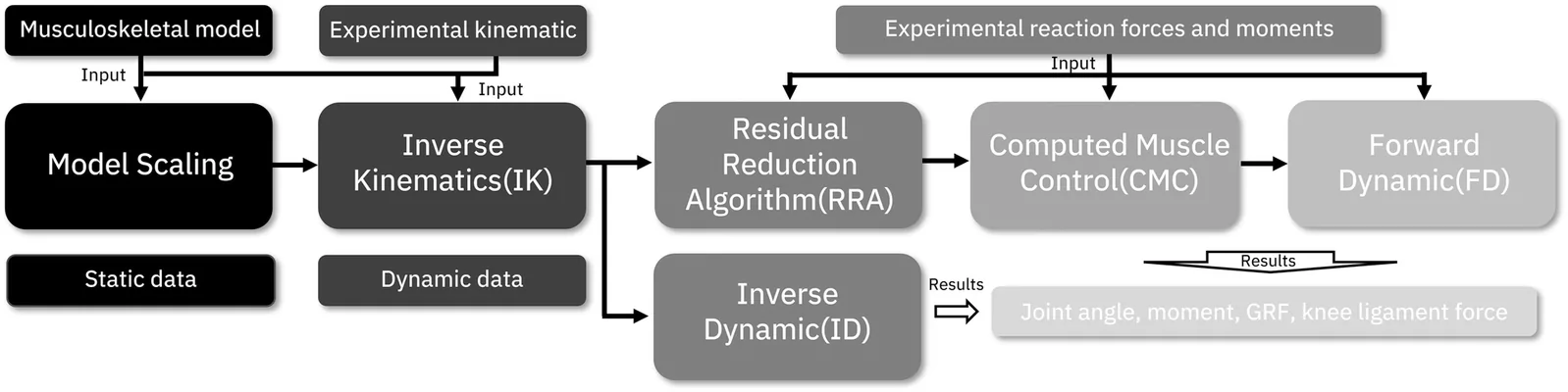
Standard openSim data processing workflow for knee ligament force estimation. The flowchart outlines the sequential pipeline from data inputs including the musculoskeletal model, static/dynamic experimental kinematics, and reaction forces/moments through core simulation stages: Model Scaling, Inverse Kinematics (IK), Residual Reduction Algorithm (RRA), Computed Muscle Control (CMC), and Forward Dynamics (FD).

Knee joint data were processed using a standard OpenSim workflow (Figure 3), which included body scaling, inverse kinematics, inverse dynamics, the residual reduction algorithm (RRA), computed muscle control (CMC), and forward dynamics (29, 42). First, each participant's anthropometric measurement was applied for body scaling to adjust segment dimensions and inertial properties. Inverse kinematics was then performed using marker trajectories to compute joint angles that matched the experimental marker data.

Inverse dynamics was used to estimate joint forces and moments based on the inverse-kinematics solutions. The residual reduction algorithm (RRA) was then applied to minimize dynamic inconsistencies between inverse kinematics and GRF data, with RRA residuals classified by OpenSim as “good”, “moderate” or “bad” according to established thresholds (42). “Good” residuals were defined as 0–10 N (0%–1.36% BW) for forces and 0–50 Nm (0%–6.8% BW) for moments residuals, whereas “moderate” residuals ranged from 10 to 25 N and 50–75 Nm for forces and moments, respectively. Residual values exceeding these thresholds were considered as “bad” (42). Table 2 represents the mean root-mean-squared (RMS) residuals and their corresponding quality classifications for each degree of freedom between the pelvis and ground. CMC was subsequently used to estimate individual muscle excitations based on maximum muscle-force-generating properties and measured motion and force data. These excitations were then applied in forward-dynamic simulation to calculate the individual ligament length and corresponding ligament forces (42).

**Table 2:** Mean of root-mean-squared residual forces(N) and moments(Nm) produced during RRA in OpenSim.

		Foot strike pattern		
		FFS	MFS	RFS
Force (N)	X	3.25 ± 7.29 (good)	2.69 ± 8.79 (good)	2.42 ± 7.00 (good)
	Y	11.77 ± 1.42 (moderate)	8.18 ± 4.11 (good)	10.12 ± 2.82 (moderate)
	Z	4.69 ± 3.69 (good)	5.17 ± 1.52 (good)	3.33 ± 4.56 (good)
Moment (Nm)	X	20.41 ± 9.58 (good)	18.14 ± 3.57 (good)	15.02 ± 7.53 (good)
	Y	9.44 ± 2.75 (good)	11.90 ± 3.74 (good)	12.34 ± 4.24 (good)
	Z	13.89 ± 3.40 (good)	8.03 ± 3.72 (good)	9.28 ± 3.12 (good)

FFS, Forefoot strike; MFS, Midfoot strike; RFS, Rearfoot strike.

To calculate the passive forces generated by the ACL and PCL bundles, a non-linear spring-ligament formulation based on the formulations by previous studies was utilized (46). The instantaneous tensile force (f) for each ligament bundle was determined as a function of ligament strain (ϵ) as follows:

$$f=\;\{\begin{array}{cc} \frac{\frac{1}{4}k\epsilon^{2}}{\epsilon_{l}}, & 0\leq \epsilon \leq 2\epsilon_{l}\\ k(\epsilon -\epsilon_{l}), & \epsilon >2\epsilon_{l}\\ 0, & \epsilon <0 \end{array}$$

where *k* represents the linear spring-stiffness parameter determined by the maximum isometric force ($F_{0}$ reported in Table 1), and $\epsilon_{l}$ is the transition strain of the ligament. The instantaneous ligament strain (ϵ) was calculated from its instantaneous length (L) relative to the zero-load slack length ($L_{0}$, reported as resting length in Table 1) using the following equation:

$$\epsilon =\frac{L-L_{0}}{L_{0}}$$

The peak ligament force was subsequently identified as the maximum value of *f* captured during the landing phase of running. In this study, the “landing phase” was operationally defined as the period from initial contact (vertical GRF > 10 N) to the point of peak knee flexion during the stance phase, capturing the entire deceleration and braking interval. The peak ligament forces were extracted strictly within this specific time frame to evaluate maximal loading during impact attenuation. Additionally, the cumulative loading on each ligament bundle during the landing phase was quantified by calculating the ligament impulse. The ligament impulse (Ns/BW) was calculated by integrating the ligament force curve over time from initial contact to the point of peak knee flexion.

### Statistical analyses

The normality of all variables was verified using a one-sample Shapiro–Wilk test (*p* > .05), confirming that the data satisfied the assumptions for parametric statistical testing. Kinetic variables were analyzed with a one-way repeated measure ANOVA, and *post-hoc* pairwise comparisons were conducted with Bonferroni correction. Effect sizes were calculated as Cohen's *d*. To evaluate the mechanical correlation between ground reaction force characteristics and knee ligament loading, Pearson correlation analyses were performed. Specifically, the correlations between the vertical GRF and ligament peak forces, as well as between the vertical GRF and ligament impulses, were analyzed across the three different foot strike types. For these Pearson correlation analyses, participant averaged data were utilized within each footstrike condition as the primary analysis unit. All statistical analyses were performed in SPSS (version 23.0, IBM Corp., Armonk, NY, USA), with the significance level set as *α* = 0.05, and exact *p*-values were reported for the correlation results.

## Results

### Conventional kinematic and kinetic variables

The ANOVA (Table 3) revealed significant differences in contact time (*F* = 6.55, *p* = .028), with *post-hoc* test indicating that FFS exhibited a shorter contact time than RFS (*p* = .024). Larger knee flexion was observed in RFS than FFS (*F* = 7.26, *p* = .005). For kinetic variables, a significant difference was observed in knee extension moment (*F* = 4.76, *p* = .030), where FFS generated a larger extension moment than RFS (*p* = .044). Vertical GRF was significantly higher in FFS compared with RFS (*p* = .027).

**Table 3:** The results of running parameters, kinetic variables as foot strike type.

	Foot strike pattern			*F*	*p*	Post hoc
	FFS	MFS	RFS			
Spatiotemporal parameters						
Cadence (steps/min)	183.60 ± 15.22	177.65 ± 20.88	171.23 ± 19.52	1.622	.210	
Contact time (s)	0.18 ± 0.02	0.20 ± 0.01	0.21 ± 0.02	6.549	.028*	FFS > RFS
Step length (m)	1.42 ± 0.14	1.44 ± 0.13	1.42 ± 0.20	0.238	.846	
Knee joint angle (deg)						
Flexion	42.06 ± 3.98	44.81 ± 2.60	51.03 ± 3.69	7.261	.005*	FFS < RFS
Adduction	9.51 ± 5.33	4.61 ± 8.11	6.91 ± 8.92	0.891	.348	
External rotation	−13.31 ± 7.80	−12.42 ± 5.15	−13.22 ± 6.85	0.157	.906	
Knee joint moment [Nm/(BW × ht)]						
Extension	3.93 ± 0.42	3.46 ± 0.30	3.33 ± 0.41	4.755	.030*	FFS > RFS
Abduction	1.65 ± 0.65	1.46 ± 0.53	1.53 ± 0.81	0.625	.420	
Internal rotation	1.22 ± 0.48	1.24 ± 0.56	1.20 ± 0.61	0.583	.484	
Ground reaction force (N/BW)						
Vertical impact peak	2.83 ± 0.27	2.75 ± 0.33	2.39 ± 0.25	4.447	.035*	FFS > RFS

FFS, forefoot strike; MFS, midfoot strike; RFS, rearfoot strike.

* *p* < .05.

### Knee ligament force & impulse

**Figure 4:**
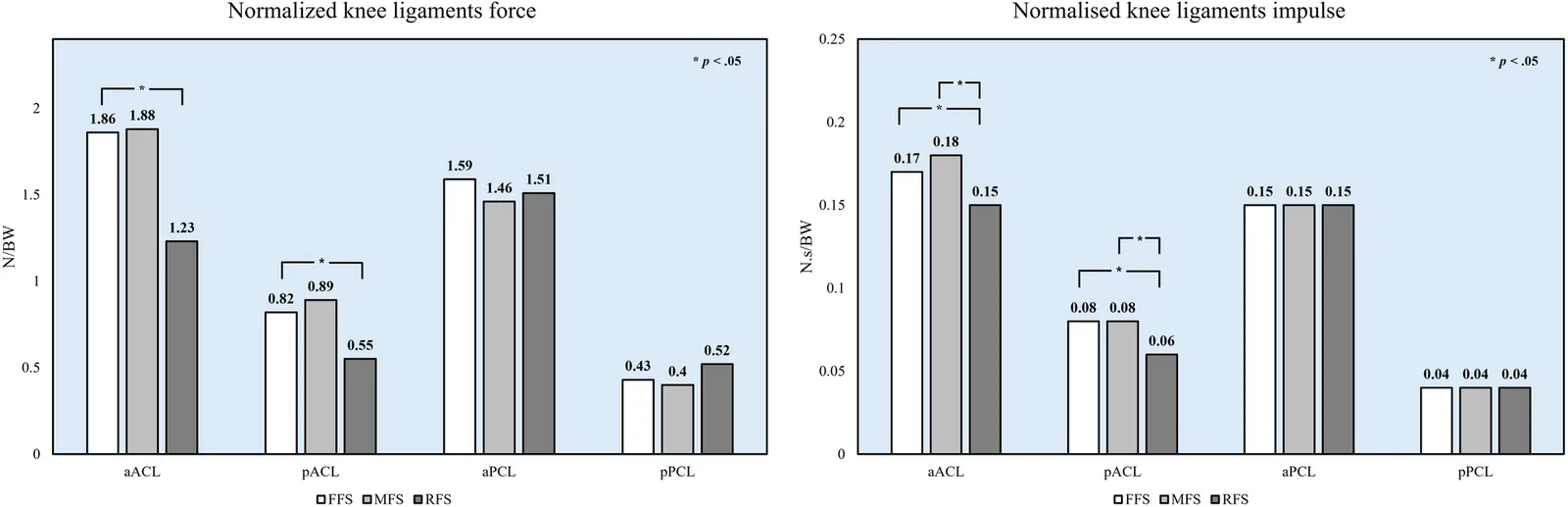
Comparison of body weight-normalized peak knee ligaments force (left, in BW) and impulses (right, in N·s/BW) across three running footstrike patterns (FFS, MFS, and RFS).

The ANOVA (Table 4) revealed significant differences in anterior and posterior ACL forces during foot contact (aACL: *F* = 6.61, *p* = .008, pACL: *F* = 6.15, *p* = .009). *Post-hoc* analyses revealed that both anterior and posterior ACL forces were significantly lower in RFS compared with FFS and MFS (*p* = .008) (Figure 4). The anterior and posterior ACL impulses showed statistically significant differences depending on the foot strike type (aACL: *F* = 83.89, *p* < .001; pACL: *F* = 141.00, *p* < .001). *Post-hoc* analyses indicated that the aACL impulse and pACL impulse in RFS were significantly lower than those in FFS and MFS (*p* < .05). In contrast, no statistically significant differences were observed across the foot strike patterns for both peak force and impulse in aPCL and pPCL.

**Table 4:** The results of peak ACL and PCL forces and impulse by foot strike type.

	Foot strike pattern			*F*	*p*	Post hoc
	FFS	MFS	RFS			
Peak ligaments force(N/BW)						
aACL	1.86 ± 0.17	1.88 ± 0.23	1.23 ± 0.31	6.605	.008*	FFS > RFS
pACL	0.82 ± 0.29	0.89 ± 0.20	0.55 ± 0.09	6.152	.009*	FFS > RFS
aPCL	1.59 ± 0.46	1.46 ± 0.32	1.51 ± 0.33	0.349	.707	
pPCL	0.43 ± 1.02	0.40 ± 0.90	0.52 ± 0.78	0.089	.915	
Ligaments impulse(N·s/BW)						
aACL	0.17 ± 0.01	0.18 ± 0.01	0.15 ± 0.01	83.889	**<**.001*	FFS > RFS
						MFS > RFS
pACL	0.08 ± 0.01	0.08 ± 0.01	0.06 ± 0.01	141.003	**<**.001*	FFS > RFS
						MFS > RFS
aPCL	0.15 ± 0.02	0.15 ± 0.01	0.15 ± 0.02	.844	.437	
pPCL	0.04 ± 0.01	0.04 ± 0.01	0.04 ± 0.01	1.933	.162	

FFS, forefoot strike; MFS, midfoot strike; RFS, rearfoot strike.

* *p* < .05.

### Pearson correlation analysis between vertical GRF and ligament peak forces, and between vertical GRF and ligament impulses

The Pearson correlation analysis results (Table 5) showed that the relationships between vertical GRF and knee ligament loadings varied depending on the foot strike type. In the case of the ACL, the vertical GRF demonstrated statistically significant, moderate positive correlations with the peak forces of both the aACL and pACL across all foot strike patterns (FFS: *r* = .627, *p* = .003 and *r* = .600, *p* = .005; MFS: *r* = .650, *p* = .002 and *r* = .641, *p* = .002; RFS: *r* = .583, *p* = .007 and *r* = .558, *p* = .010, respectively). Furthermore, positive correlations were observed between the vertical GRF and ACL impulses under all footstrike types (FFS: *r* = .821, *p* < .001 and *r* = .804, *p* < .001; MFS: *r* = .845, *p* < .001 and *r* = .827, *p* < .001; RFS: *r* = .778, *p* < .001 and *r* = .735, *p* < .001, respectively). In contrast, no meaningful correlations were found between the vertical GRF and any of the aPCL and pPCL.

**Table 5:** Pearson correlation analysis between vertical GRF and ligament peak forces, and between vertical GRF and ligament impulses by foot strike type.

	Foot strike pattern					
	FFS		MFS		RFS	
	*r*	*p*	*r*	*p*	*r*	*p*
Vertical GRF and ligament peak force						
aACL	0.627	.003**	0.650	.002**	0.583	.007**
pACL	0.600	.005**	0.641	.002**	0.558	.010*
aPCL	0.052	.827	−0.021	.930	0.030	.828
pPCL	−0.049	.837	0.040	.867	−0.018	.838
Vertical GRF and ligament impulse						
aACL	0.821	**<**.001***	0.845	**<**.001***	0.778	**<**.001***
pACL	0.804	**<**.001***	0.827	**<**.001***	0.735	**<**.001***
aPCL	0.020	.933	−0.049	.834	0.036	.867
pPCL	−0.028	.900	0.021	.933	−0.021	.933

FFS, forefoot strike; MFS, midfoot strike; RFS, rearfoot strike.

* *p* < .05.

** *p* < .01.

*** *p* < .001.

## Discussion

Using a musculoskeletal model, the present study examined the internal knee-ligament loading along with conventional kinematic and kinetic variables across three running footstrike patterns at the same controlled speed. Forefoot striking produced higher vertical GRF and larger knee extension moments, coinciding with increased ACL loading compared with the other two patterns. These observations partly agree with our prior expectations based solely on the external joint kinematics and kinetics, which often hypothesize that non-RFS patterns shift loading distally to the Achilles, plantar fascia, and tibia (7). The increased ACL loading would require further investigation into how the loading transfers from the ground to the knee via lower limb kinetic chain.

Contact time was shorter in FFS than other two footstrike patterns, consistent with the previous studies (1, 47). Shorter contact time would concentrate forefoot pressure over the fifth metatarsal (48) and elevate localized pressure by two to three times relative to the more distributed loading seen in MFS and RFS, potentially contributing to greater muscle fatigue with prolonged running (49). FFS also increased gastrocnemius activation, which may further elevate Achilles tendon loading (35, 50), and fatigue-related increases in forefoot stress may raise fracture risk (50, 51). Although our study did not measure plantar pressure, future work should incorporate center-of-pressure trajectories and pressure-time integrals to ascertain relationship among footstrike pattern, localized loading, and fatigue.

In our study, knee flexion angle at initial contact was greater in RFS than other two footstrike patterns. This is associated with improved impact attenuation (28, 52). The longer contact time observed in RFS allow greater opportunity for knee flexion for attenuating GRF impact (23, 53). Better attenuation of rapid loading has been linked to smaller frontal-plane body sway for improved knee stability (52) and lower injury risks (1, 54). Most notably, as detailed in Table 3, FFS exhibited the shortest contact time (0.18 s) among all conditions, which limits the capacity to temporally disperse the impact forces transmitted to the body (55, 56). This shortened duration forces the higher vertical GRF and greater knee extension moments to be rapidly transferred into the lower limb joints, thereby mechanically driving the acute increase in ACL loading. In contrast, both FFS and MFS exhibited smaller knee flexion angles with higher vertical GRF peaks and larger knee extension moments. FFS typically presents greater plantarflexion at contact, positioning the GRF vector more anterior to the ankle joint (57). MFS has been associated with higher impact forces than RFS in some extents. Reduced impact attenuation at midfoot striking may transmit impact more directly through the lower limbs, potentially increasing tissue stress (58). Although MFS may offer certain biomechanical advantages, our results reveal less effective attenuation impact than RFS, underscoring the need to consider impact management in gait re-training program. Moreover, an anterior shift of the GRF vector can increase gastrocnemius and soleus activity, creating a larger dorsiflexion moment and potentially increasing joint loading upstream (23, 59). Taken together with prior literature, FFS may be less suitable for prolonged running due to higher muscular demand and energetic cost as well as associated changes in internal joint loading profiles (1). However, these findings should be interpreted strictly as modeled ligament loading under the specific straight-line running conditions tested, rather than direct evidence of macro-injury risk. Our GRF results differ from some previous RFS observations (60). Such discrepancies may reflect differences in speed, footwear, surface, coaching of footstrike patterns, sample characteristics, filtering choices, or the specific variables reported. Injury risk and mechanisms are context-dependent; Future studies should integrate these factors for a more comprehensive understanding.

From a ligament-level perspective, our results indicated greater forces in the anterior and posterior ACL bundles in FFS. A plausible mechanism is that FFS supports body load over a smaller contact area whilst maintaining a more extended knee posture, which may increase activation of ankle and knee extensors (61). With the knee closer to extension, ligament tensile forces can rise more rapidly (22, 61). During FFS, early body deceleration occurs with an extended knee, whereas RFS and MFS exhibit comparatively greater knee flexion at contact. At the extended-knee posture, strong quadricep contraction is required to elevate ACL loading by increasing anterior tibial shear (15, 62, 63). The observed moderate-to-strong positive correlations between vertical GRF and ACL loading (peak and impulse) suggest that impact magnitude may be an important contributor to ACL loading during running. However, the lack of significant correlations for the PCL indicates that ACL loading may be more sensitive to impact-related loading mechanisms, whereas PCL loading is influenced by other biomechanical factors.

Most ACL loading studies have focused on cutting and change-of-direction tasks, where injury thresholds can exceed twice body weight (29, 64). Our work provides ligament-loading profiles across footstrike pattern during straight-line running, as it is a fundamental component in most sports. It is crucial to note that non-contact ACL ruptures occur during high-risk, multi-planar maneuvers such as cutting, pivoting, rapid deceleration, or jump landings, rather than steady-state linear running. Therefore, our results do not imply that RFS is universally safer or that FFS necessarily elevates clinical ACL injury risk; instead, they reflect specific variations in modeled ligament loading under the tested conditions. If cutting or pivoting occurs after prolonged running, accumulated fatigue may elevate ligament loading and thus injury risk (65). These results emphasize the value of technique, particularly knee flexion at contact and impact attenuation in injury prevention and rehabilitation. For some runners, adopting a MFS may reduce localized heel loading by increasing plantar contact area at initial contact (66) and greater arch motion during MFS may promote more uniform pressure distribution (67). The optimal strategy should be individualized and balanced against trade-offs at the ankle-foot complex.

In this study, ACL loading was significantly lower in RFS than in FFS and MFS, whereas PCL did not differ significantly among patterns. Although ACL and PCL ruptures rarely occur during straight-line running and are more common during lateral and rotational maneuvers, previous work suggest that RFS may increase the risk of other conditions (e.g., tibial fractures, patellofemoral pain, and knee osteoarthritis) due to higher loading rates in some contexts (68–70). Nonetheless, our ligament-level results can inform rehabilitation planning; selecting a footstrike that increases knee flexion at contact and enhances impact attenuation may help reduce repetitive ACL tension during return-to-run period. Long-term risks should be evaluated holistically, considering potential trade-offs elsewhere in kinetic chain.

Some limitations should be considered when interpreting these results. First, this study used a single footwear model, and variations in midsole thickness or cushioning properties could influence natural footstrike patterns (71). Second, the three footstrike patterns were experimentally imposed rather than reflecting the participants' habitual running patterns or training history. Thus, the ecological validity of these imposed conditions should be interpreted with caution. Third, this study included only healthy, young male adults, which limits the generalizability of our ACL-related conclusions to female athletes, ACL-reconstructed individuals, or other populations inherently at high risk of ACL injury. Finally, ligament properties were literature-based rather than subject-specific, and we did not capture plantar pressure or EMG, which could clarify foot-ankle loading and co-contraction effects. Future study should test multiple running speeds and ecologically valid conditions, incorporating EMG-informed modeling and subject-specific anatomy for ligament parameters.

## Conclusion

Forefoot striking produced higher ACL loading associated with higher vertical GRF and greater knee extension moments than rearfoot striking, whereas rearfoot striking was characterized by greater knee flexion and lower ACL forces. These findings indicate that distinct footstrike patterns alter internal tissue-level mechanical environments, suggesting that modifications in footstrike mechanics may modulate acute ligament loading during the stance phase of running. Since these simulated results are context-dependent and limited to steady-state straight-line running, they should be interpreted cautiously rather than as direct clinical indicators for injury prevention. Conversely, higher ACL loads associated with FFS were observed under the tested straight-line running condition. Understanding these ligament mechanics could provide valuable insights for future research on injury-prevention strategies and rehabilitation protocols. Future research incorporating long-term prospective training designs is warranted to investigate the holistic effects of footstrike retraining across varied speeds, surfaces, and footwear conditions before these tissue-level metrics can be safely integrated into clinical return-to-play decision-making frameworks.

## Data Availability

The raw data supporting the conclusions of this article will be made available by the authors, without undue reservation.
